# Supplementary material for: Evidence of hatch‐time based growth compensation in the early life history of two salmonid fishes
Source: Ecol Evol. 2022 Dec 15;12(12):e9636. doi: 10.1002/ece3.9636 (PMC9755820; doi:10.1002/ece3.9636)
Supplement: Supplementary file 1 — Appendix S1. [file ECE3-12-e9636-s001.docx]

Appendices:

**Appendix A1**: Fish sampling locations (degrees decimal minutes) and temperature logger locations sampled June 24 to June 29^th^, 2013, in northern Labrador, Canada. Number of loggers at each location included in brackets. (TL = locations with temperature loggers).

| **Site** | **River name** | **Sampled** | **Coordinates** | | **Logger location** |
| --- | --- | --- | --- | --- | --- |
|  |  |  | **Latitude** | **Longitude** |  |
| 1 | Hebron River | Yes | 57°N 51.96 | 63°W 32.37 |  |
| 3 | Kamanatsuk Brook | Yes | 56°N 45.48 | 62°W 52.31 |  |
| 2 | Fraser River | Yes | 56°N 42.34 | 63°W 32.90 (TL) | Spawning bed (2) |
| 4 | Anaktalik Brook | Yes | 56°N 29.94  52°N 30.03  56°N 30.01 | 62°W 55.68 (TL)  62°W 56.79  62°W 55.37 | River (1) |
| 6 | Ikadlivik Brook | No | 56°N 24.00 | 62°W 31.55 (TL) | River (1) |
| 5 | Igluvigaluk Brook | Yes | 56°N 17.43  56°N 17.64  56°N 16.64 | 62°W 23.86  62°W 23.58  62°W 26.06 |  |


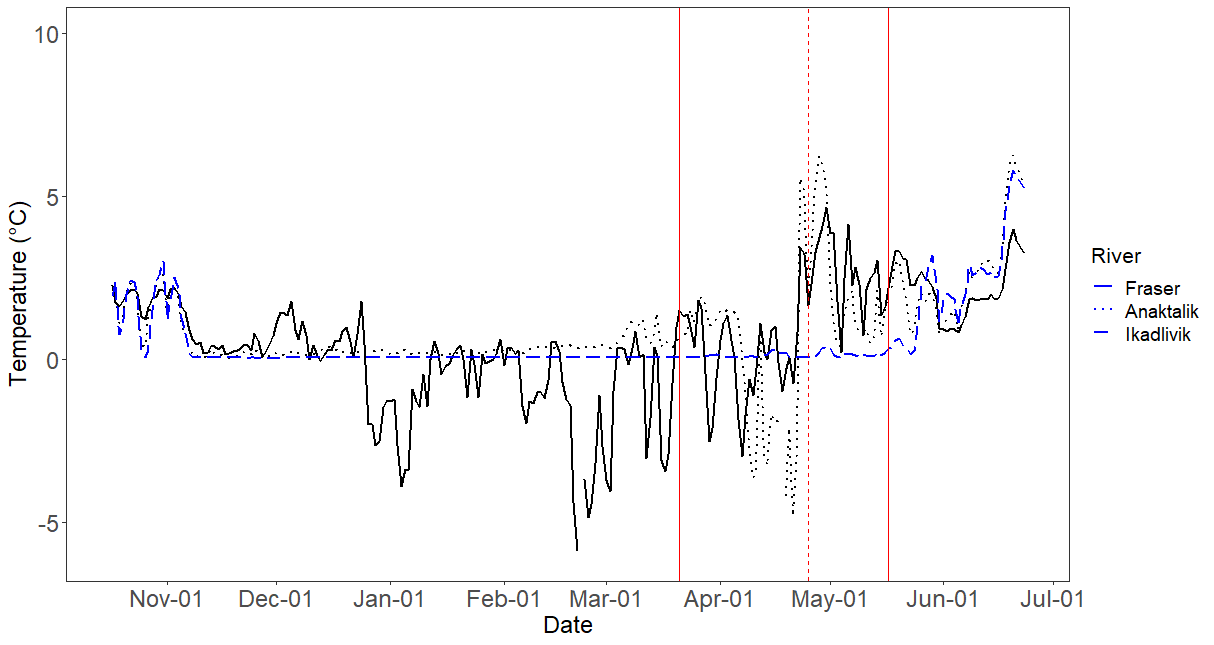

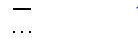


**Appendix A2.** Temperature profiles from Fraser (black line) (average of 2 loggers), Ikadlivik (blue, dashed line) and Anaktalik (black, dotted line) rivers in Labrador, Canada from loggers in place from October 2012 to June 2013. Vertical lines indicate hatching dates for both species and all rivers (solid=range, dashed= mean). Note: negative values likely indicate being frozen in ice.


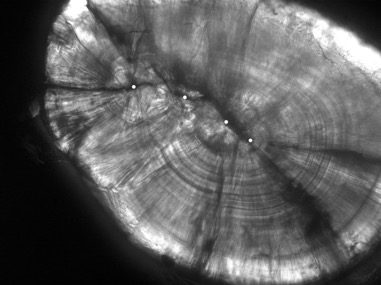


**A**


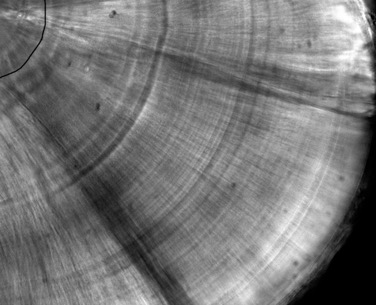


**B**

**H**

**Appendix A3**. Example photographs of otoliths from two Arctic charr sampled from Anaktalik River, Labrador taken under a compound microscope and then manipulated in Photoshop. A) A whole salmonid otolith (40x) with hatch line (white arrow) and B) a close-up photo (100x, under oil immersion), with hatch (H, black line) and emergence (white arrow) checks indicated. White dots indicate primordia.

**B**

**Appendix A4:** Total number of Arctic charr and brook trout (*Salvelinus alpinus* and *S. fontinalis*) for each river caught June 24 to June 29^th^, 2013 in Northern Labrador, Canada and subsequently aged. Only species that had data on more than 10 individuals in a river were included in the analyses.

|  |  | **Anaktalik** | **Fraser** | **Hebron** | **Kamanatsuk** | **Igluvigaluk** | **Total** |
| --- | --- | --- | --- | --- | --- | --- | --- |
| **Arctic charr** | Caught  Aged | 100  92 | 40  19 | 107  83 | 3  0 | 18  12 | 268  206 |
| **Brook trout** | Caught  Aged | 5  0 | 3  0 | 4  0 | 129  100 | 27  18 | 168  118 |
| **Total** | Caught  Aged | 105  92 | 43  19 | 111  83 | 132  100 | 45  30 | 436  324 |
